# Mesenchymal Stromal Cells: Novel Methods for Characterization, Understanding Differentiation, and Function

**DOI:** 10.1155/2014/630936

**Published:** 2014-04-09

**Authors:** Vivek Tanavde, Mohan C. Vemuri, Radhika Pochampally

**Affiliations:** ^1^Bioinformatics Institute, Agency for Science Technology and Research (A∗STAR), Singapore 138671; ^2^Thermo Fisher Scientific, Frederick, MD 21704, USA; ^3^Cancer Institute, University of Mississippi Medical Center, Jackson, MS 39216, USA

MSCs are increasingly being used to treat a wide variety of disorders. These include diseases of the musculoskeletal system, wound healing, and vascular disorders. The tissue sources for MSCs are also growing. In addition to the traditional bone marrow and adipose tissue, many adult tissues have resident MSC populations that contribute to the renewal and replacement of cells in that tissue. Tissues like umbilical cord/Wharton's jelly, dental pulp, peripheral muscle, and so forth are also gaining acceptance as viable sources of MSCs.

The International Society for Cellular Therapy (ISCT) adopted a definition for MSCs in 2006. MSCs defined by these markers are not a homogenous cell population and exhibit tissue specific differences. Increasingly, MSCs are being used in a variety of veterinary applications using autologous transplantation. Therefore, there is an urgent need for complete characterization of the animal MSCs. Similar to human MSCs, animal MSCs clinical trials are hindered by lack of well-defined antibodies in different animal species for isolation and identification of those cells. Therefore, it is very important to develop new methods for MSC characterization for veterinary use. Thus, the challenges of comprehensively defining MSCs from different tissues and different species remain. The main hurdles to overcome for routine therapeutic use of MSCs have been in vitro expansion of cells (1) while retaining multipotentiality, (2) while maintaining the epigenetic and genetic stability of cells, and (3) while obtaining therapeutically effective numbers of cells efficiently.

Although MSCs have been reported to differentiate into a wide variety of different cell types in vitro, it has been a challenge to generate enough differentiated cells from MSCs for therapeutic applications. This is especially true since MSCs are differentiating into lineages other than bone, cartilage, or fat. Thus, it is important to generate protocols for the massive expansion of native MSCs that can be differentiated efficiently into different lineages or protocols that achieve massive expansion in cell number during differentiation into a specific lineage. The articles in this special issue address some of these challenges while providing some interesting solutions.

Transcriptome analysis of MSCs from different sources is increasingly revealing the heterogenous nature of MSCs derived from different sources. Although these MSCs are all based on surface marker expression, their gene expression profiles are quite different. The paper by J. Bosch et al. reports similar findings when comparing gene expression profiles of bone marrow versus cord blood MSCs. These results may have important implications in defining markers for MSC. It is possible that we need a different set of gene markers for MSCs based on their origin.

Genetic and epigenetic stability of MSCs during culture has been another concern during expansion of cells to obtain the high number of cells for therapeutic applications. The review article by A. Bentivegna et al. comprehensively summarizes the knowledge of genomic and epigenomic stability of MSCs during extended culture periods. The authors summarize that MSC genome is extremely stable during extended periods of culture, perhaps aided by methylation of specific gene promoters.

Another challenge for therapeutic use of MSCs is efficiently generating large numbers of MSCs for specific applications. In their article, P. Prasajak and W. Leeanansaksiri describe a highly efficient protocol for hepatocyte differentiation of Wharton's jelly derived MSCs in just 18 days. Using hypoxic conditions, the authors were able to achieve 80% efficiency of conversion of MSCs to hepatocytes which is quite remarkable. The review by C. M. Raynaud and A. Rafii further elaborates on the characterization necessary for clinical use of MSCs. The authors raise pertinent questions like what is the definition of cells being used for therapy, whether MSCs from different tissues are equivalent, what is the context in which MSCs should be used for clinical trials and so forth. It is important to answer these questions in a systematic manner before widespread use of MSCs in the clinic becomes accepted.

The last two articles in this issue focus on the use of surface markers in isolation of MSCs from different animal species. The review article by Y. Mabuchi et al. summarizes the marker expression of murine and human MSCs. They propose the use of PDGF-R*α* and Sca-1 as markers for prospective isolation of murine MSCs. The article by C. Adamzyk et al. describes the effect of culture media on sheep MSCs. They conclude that variation in culture media combined with biological variation between different animals introduces huge variations in the gene expression profiles of MSCs. This makes it difficult to identify reliable biomarkers for sheep MSCs from gene expression data. Such variations in culture conditions may also have important implications for the downstream function of MSCs.

We hope these latest original research articles in the field of MSC research would promote and enable better understanding of the basic biology of MSC that is critical to the successful use of these cells in patient specific translational therapies.

*Vivek Tanavde* *Vivek Tanavde* *Mohan C. Vemuri* *Mohan C. Vemuri* *Radhika Pochampally* *Radhika Pochampally*

